# Biomechanical effects of joint disc perforation on the temporomandibular joint: a 3D finite element study

**DOI:** 10.1186/s12903-023-03521-x

**Published:** 2023-11-29

**Authors:** Wentong Gao, Jie Lu, Xiang Gao, Jianping Zhou, Hongwei Dai, Min Sun, Jie Xu

**Affiliations:** 1 Stomatological Hospital of Chongqing Medical University, No. 426 Songshi North Road, Chongqing, China; 2 Chongqing Key Laboratory for Oral Diseases and Biomedical Science, Chongqing, China; 3 Chongqing Municipal Key Laboratory of Oral Biomedical Engineering of Higher Education, Chongqing, China; 4 Department of Orthopedics, The First Affiliated Hospital of Chongqing Medical University, Chongqing, China; 5 Department of Knee Joint Sports Injury, Sichuan Provincial Orthopedic Hospital, Chengdu, Sichuan Province China

**Keywords:** Finite element method (FEM), Biomechanics, Temporomandibular joint disorder (TMD), Disc displacement, Disc perforation (DP)

## Abstract

**Background:**

Disc perforation (DP) is a severe type of Temporomandibular Disorder (TMD). DP may induce changes in the internal stresses of the temporomandibular joint (TMJ). Herein, this study attempts to investigate the biomechanical effects of different positions and sizes of DP on the TMJ using a biomechanical approach, to explore the mechanical pathogenesis of TMD.

**Methods:**

Eleven three-dimensional finite element (FE)models of the TMJ were constructed based on CBCT imaging files of a patient with DP on the left side. These models included the disc with anterior displacement and discs with different locations and sizes of perforations on the affected disc. FE methods were conducted on these models.

**Results:**

Anterior displacement of the disc leads to a significant increase in the maxim von Mises stress (MVMS) in both TMJs, with the affected side exhibiting a more pronounced effect. DP occurring at the posterior band and the junction between the disc and the bilaminar region has a greater impact on the MVMS of both TMJs compared to perforations at other locations. As the size of the perforation increases, both sides of the TMJs exhibit an increase in the magnitude of MVMS.

**Conclusions:**

Unilateral disc anterior displacement results in an increased stress on both TMJs. Unilateral DP further affects the stress on both sides of the TMJs. TMD is a progressive condition, and timely intervention is necessary in the early stages to prevent the worsening of the condition.

## Background

The temporomandibular joint (TMJ) is the only bilateral synovial joint in the human body. The TMJ consists of the temporal bone’s glenoid fossa and articular eminence, the mandibular condyle, the disc, the joint capsule, and the intra- and extra-capsular ligaments [1, 2]. The disc is located between the condyle and the glenoid fossa. The cross-section of the disc is S-shaped. This arrangement prevents structural damage by direct contact between the condyle and the temporal bone’s glenoid fossa. Moreover, the disc acts as a cushion, mitigating mechanical pressure and facilitating coordinated movements of the condyle [3, 4].

Disc perforation (DP) is a severe type of Temporomandibular Disorder (TMD), that predominantly occurs at the posterior band of the TMJ disc, the bilaminar region, and the junction of the posterior band with the bilaminar region [5, 6]. The main clinical manifestations of DP include impaired jaw movement, continuous friction sound within the joint, and pain in the joint area during the opening and closing of the mouth. DP can also be accompanied by damage to other soft and hard tissues of the joint, often leading to pathological changes in the articular cartilage, subchondral bone, and ligaments. These conditions significantly impact the physiological and psychological well-being of affected patients [7].

DP affects the morphology and structure of the TMJ, often leading to changes in the internal stress environment of the TMJ [8, 9]. Currently, conservative treatment remains the primary approach for disc perforation, with surgical intervention often considered a supplementary option when conservative treatment fails. However, the indications for disc perforation surgery are not well-defined, resulting in significant differences of opinion among joint specialists regarding whether surgery should be performed. Therefore, research on the biomechanics of the TMJ after disc perforation is crucial.

The finite element method (FEM) is a numerical simulation method that has proven effective in solving complex structural mechanics problems [10, 11]. In recent years, FEM has been widely applied in the field of TMJ biomechanics. Using FEM, Abe et al. have demonstrated that after disc displacement, the primary load-bearing area of the TMJ shifts posteriorly, accompanied by an increase in the maximum stress borne by the disc [12]. Related research by Shao et al. found that in the presence of TMD, the affected side experiences higher structural stresses compared to the normal side [13].

The primary objective of this study is to investigate the mechanical characteristics of the TMJ after DP using a 3D FE model. In this study, we collected CT images of a patient with DP on the left side of the TMJ, while the right side was in a normal state. These images were utilized to analyze the effects of different locations and sizes of DP on the TMJ following disc displacement. This approach not only provides references for determining whether patients are suitable candidates for surgery but also offers prognostic indicators for individuals with disc perforation.

## Materials and methods

### Subject and CT images

The participant in this study was a patient with DP in the left TMJ. The inclusion criteria were as follows: (1) The patient has a unilateral DP in the TMJ without any history of disease on the other side. (2) The patient does not exhibit significant malocclusion or dental misalignment. (3) The patient has no history of orthodontic treatment. (4) The patient has not undergone any restorative treatments in the oral cavity. (5) The patient has no history of maxillofacial surgical interventions.

### FE modelling

**Fig. 1:**
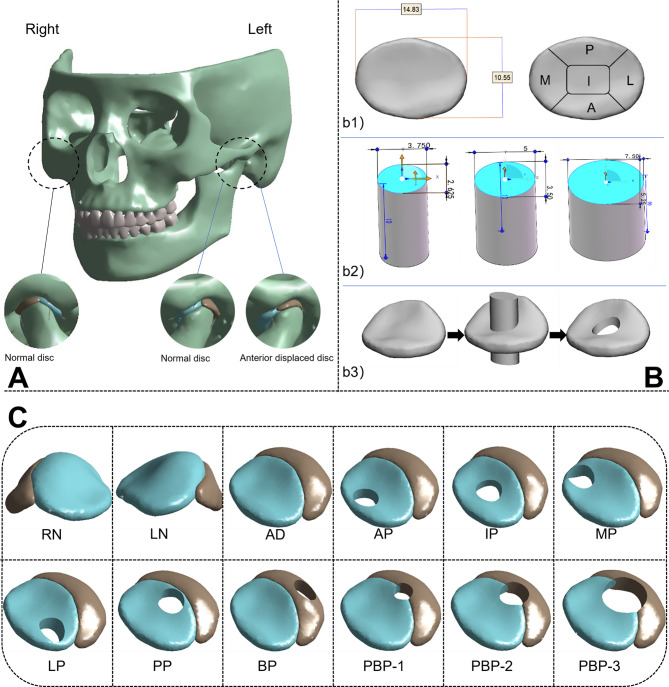
Configuration of the FEM simulations. **A**. The assembled FEM model of the skull; **B**. **b1**) The measurement and partitioning of displaced discs; **b2**) Elliptical cylinders of different sizes for perforation; **b3**) The process of building perforated discs; **C**. The FE models of TMJ discs: AP – anterior band perforated disc, IP – intermediate band perforated disc, MP – medial band perforated disc, LP – lateral band perforated disc, PP – posterior band perforated disc, BP – bilaminar region perforated disc, PBP-1 – the junction of the posterior band and bilaminar region perforated disc (1/4 of the anteriorly displaced disc), PBP-2 – the junction of the posterior band and bilaminar region perforated disc (1/3 of the anteriorly displaced disc), PBP-3 – the junction of the posterior band and bilaminar region perforated disc (1/2 of the anteriorly displaced disc)

A relatively complete 3D model of the craniofacial region was constructed by MIMICS (Materialize, Leuven, Belgium) based on DICOM image files. Since the disc and its attachments are not radiopaque in CT, the disc and bilaminar region were reconstructed based on the joint space, while the ligament attachments were simulated by 16 springs with a stiffness of 6.5 N [14, 15] [Fig. 1. A]. DP commonly occurs after disc displacement. Therefore, in addition to establishing normal discs on both sides, a disc with anterior displacement (anteroposterior diameter: 10.55 mm, inner and outer diameter: 14.83 mm) occurring on the left side was created. Based on the anteriorly displaced disc, different types of perforated discs were generated, including the anterior band perforated disc (AP), intermediate band perforated disc (IP), medial band perforated disc (MP), lateral band perforated disc (LP), posterior band perforated disc (PP), bilaminar region perforated disc (BP), and the junction of the posterior band and bilaminar region perforated disc (PBP). These models were used to investigate the effects of perforation at different locations on the TMJ. The perforation diameter was approximately 1/3 of the anteriorly displaced disc (anteroposterior diameter:3.5 mm, inner and outer diameter: 5 mm). To investigate the effects of different perforation sizes on the TMJ, perforations were made at the junction of the posterior band and bilaminar region. The perforation sizes were selected as 1/4 (anteroposterior diameter:2.625 mm, inner and outer diameter: 3.75 mm), 1/3 (anteroposterior diameter:3.5 mm, inner and outer diameter: 5 mm), and 1/2 (anteroposterior diameter:5.25 mm, inner and outer diameter: 7.5 mm) of the anteriorly displaced disc [Fig. 1. B: b1)-b3)]. The resulting model is illustrated in Fig. 1. C.

### Mesh partitioning

After smoothing and denoising procedures, the model was divided into surface meshes and volume meshes. Smaller mesh sizes can provide more accurate results but also increase the computational cost of the simulation. Therefore, based on a bone cortical mesh size of 1.25 mm and bone trabecular mesh size of 1.5 mm, further refinement of the mesh was performed at the contact areas. The mesh sizes for the condyle, articular fossa, teeth, disc, and bilaminar region were refined to 0.4 mm, 0.4 mm, 0.5 mm, 0.4 mm, and 0.35 mm, respectively.

### Material Properties

Once the mesh division was completed, assembly, computation, and final analysis of all constructed models were performed using ANSYS (ANSYS Inc, USA). In this study, the constituent materials of the TMJ were assumed to be linearly elastic and isotropic. The mechanical properties of each component are summarized in Table 1 [16].

**Table 1:** The material properties of TMJ adopted in FE models

*Tissue*	*Young’s modulus (MPa)*	*Poisson’s ratio*
Cortical bone	13,700	0.3
Cancellous bone	7930	0.3
Disc	44.1	0.4
Tooth	18,600	0.31
Bilaminar region	0.49	0.49

### Loading and boundary conditions

**Fig. 2:**
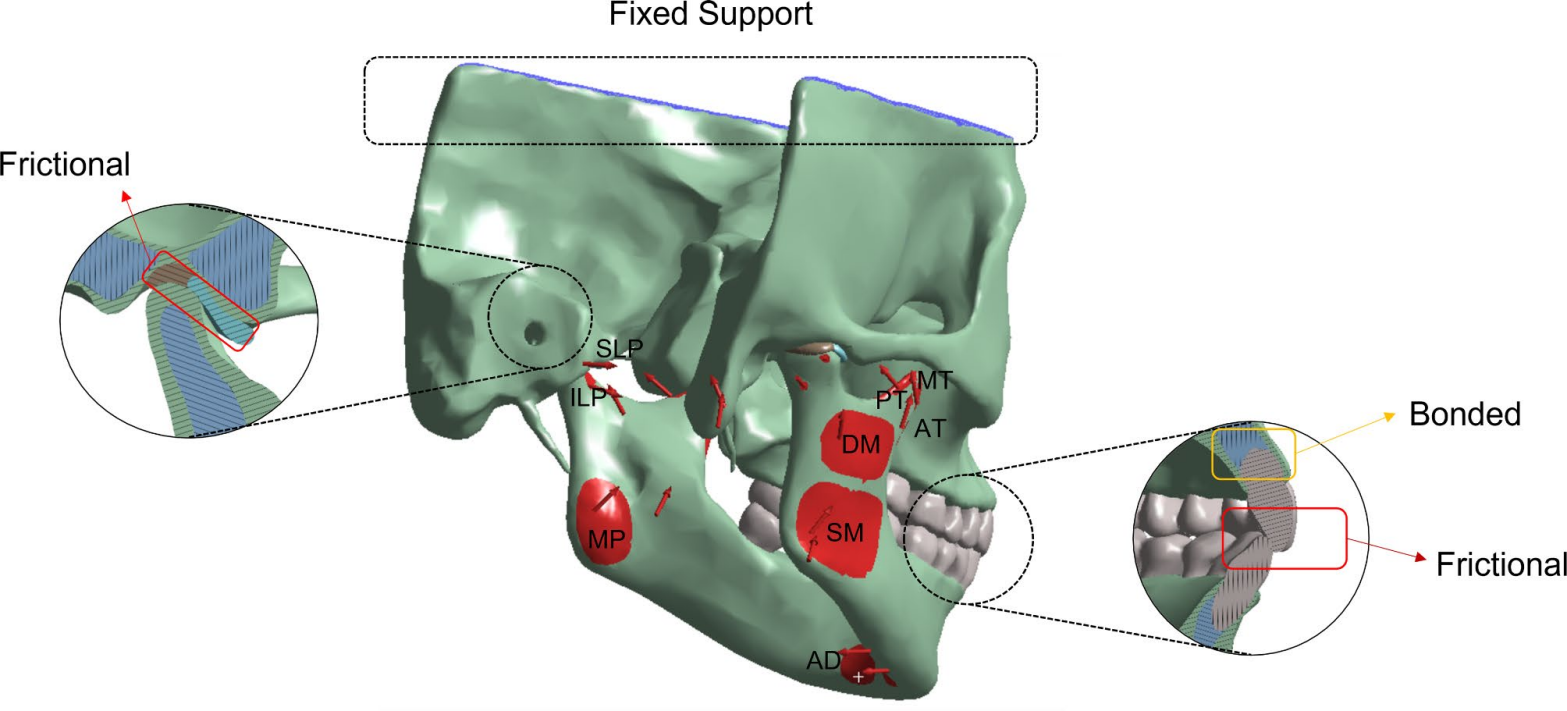
The boundary conditions of FE models. SM – Superficial Masseter, DM – Deep Masseter, AT –Anterior Temporalis, MT – Middle Temporalis, PT – Posterior Temporalis, MP – Medial Pterygoid, SLP – Superior Lateral Pterygoid, ILP– Inferior Lateral Pterygoid, AD – Anterior Digastric

This experiment employed static biomechanical analysis of the TMJ based on the intercuspal position (ICP). In our simulation, the upper part of the skull was considered fixed support to restrict the displacement of the upper model. The contact types between the disc and condyle, the disc and temporal bone joint surface, as well as between the teeth, were set as surface-to-surface contact. The contact relationships were defined as frictional contact with a coefficient of friction of 0.001 [17]. The relationships between the disc and bilaminar region, as well as between the teeth and the upper and lower jawbones, were set as fixed contacts to ensure stable contact without sliding or detachment. According to anatomical information and previous research, a total of 9 groups of muscles were included in the simulation, applying forces on the mandible [18] [Fig. 2]. Each muscle had its direction and efficiency. The maximum values and directional components of the muscle forces used in this study can be found in Table 2 [19].

**Table 2:** Forces (N) assigned to the masticatory muscles

	*Maximum muscle force (N)*	*Efficiency*	*Direction*					
			*L Cos-x*	*L Cos-y*	*L Cos-z*	*R Cos-x*	*R Cos-y*	*R Cos-z*
*SM*	190.40	1.00	39.41	-79.78	168.31	-39.41	-79.78	168.31
*DM*	81.60	1.00	44.55	29.21	61.85	-44.55	29.21	61.85
*MP*	174.80	0.76	-64.56	-49.55	105.08	64.56	-49.55	105.08
*AT*	158.00	0.98	23.07	-6.81	152.98	-23.07	-6.81	152.98
*MT*	95.60	0.96	20.37	45.89	76.82	-20.37	45.89	76.82
*PT*	75.60	0.94	14.78	60.76	33.68	-14.78	60.76	33.68
*SLP*	28.70	0.59	-12.89	-10.92	1.25	12.89	-10.92	1.25
*ILP*	66.90	0.27	-11.38	-13.67	-3.14	11.38	-13.67	-3.14
*AD*	40.00	0.28	2.73	10.53	-2.65	-2.73	10.53	-2.65

### Von Mises stress

Von Mises stress is equivalent stress based on the theory of the fourth strength, which takes into account the first, second, and third principal stresses. It is commonly used for evaluating fatigue and failure criteria. Von Mises stress is a mechanical concept in the field of elastoplastic mechanics and has been widely applied in the field of biomechanics. This study will also use it to assess the stress distribution and potential failure areas in the TMJ model.

## Result

### The impact of perforation at different locations of the disc on TMJ stress

#### Stress distribution on the discs of the affected side

**Fig. 3:**
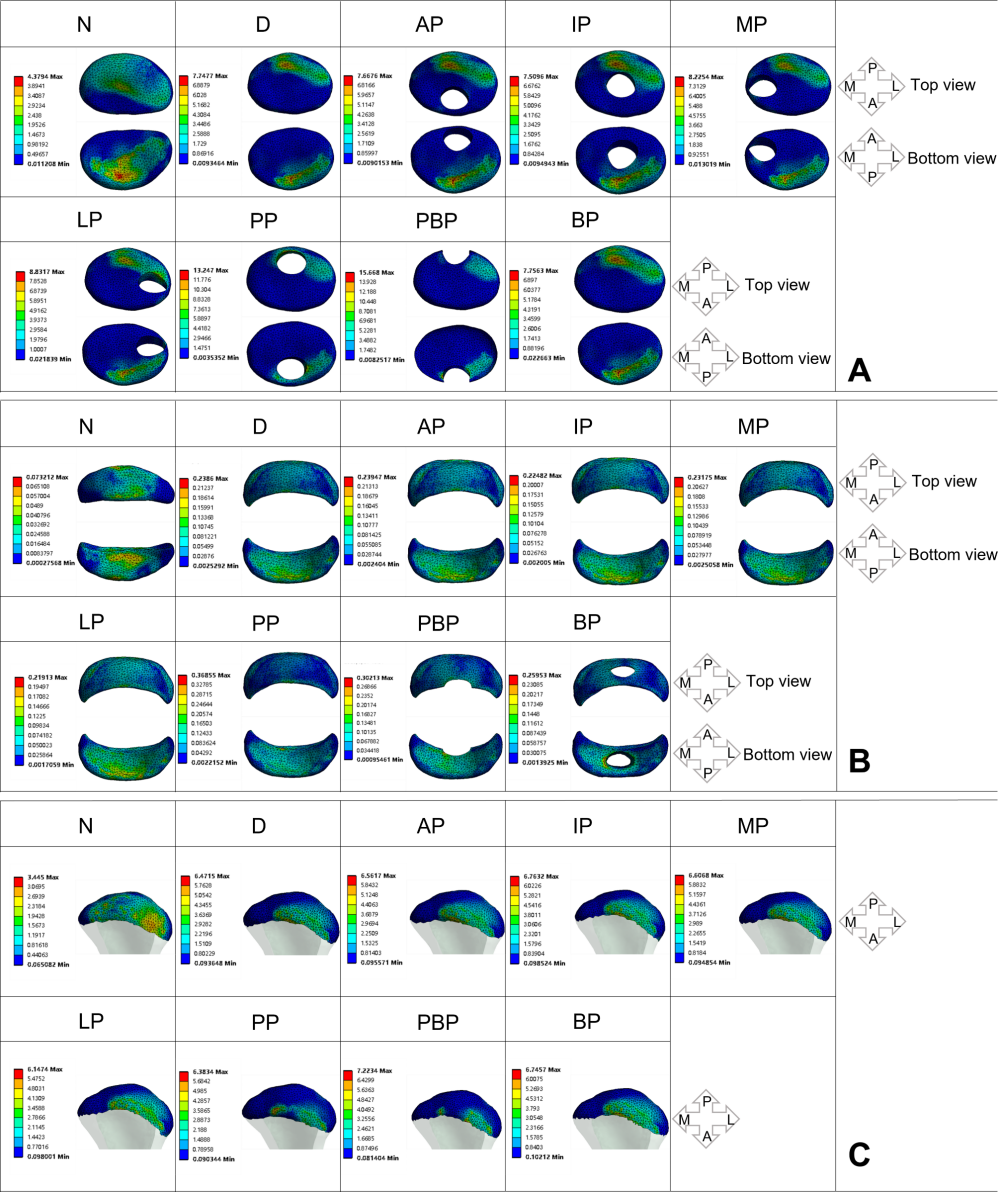
Stress distribution on affected-side TMJ impaired by disc perforated at different locations (MPa). **A**: discs, **B**: bilaminar regions, **C**: condyles

**Fig. 4:**
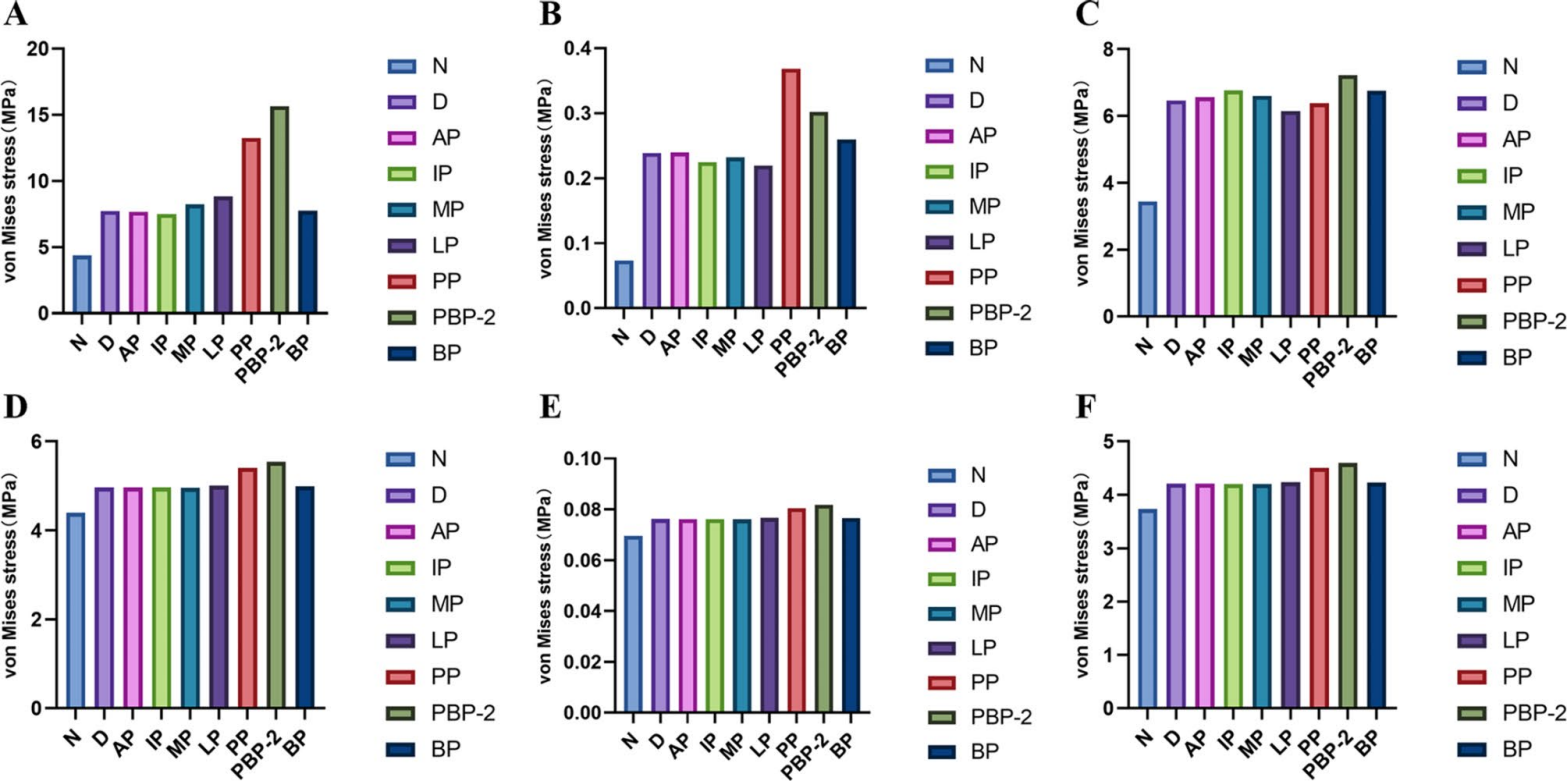
Peak value of the stress on the TMJ affected by perforated disc at different sites (MPa). **A**: affected-side discs; **B**: affected-side bilaminar regions; **C**: affected-side condyles; **D**: normal-side discs; **E**: normal-side bilaminar regions; **C**: affected-side condyles

The results of stress distribution on the discs of the affected side are shown in Fig. 3. A, B and Fig. 4. A, B. In the normal state of the affected-side disc, the maximum equivalent stress (Maximum von Mises stress, MVMS) experienced by the affected-side disc is 4.3794 MPa, while the MVMS in the bilaminar region is 0.073212 MPa. The high-stress areas in the disc are mainly distributed in the middle band and posterior band. The high-stress areas in the bilaminar region are primarily located in the anterior-central portion of the bilaminar region. After the occurrence of anterior displacement of the affected-side disc, there is a sharp increase in MVMS for both the affected-side disc and the bilaminar region, reaching 7.7477 MPa and 0.2386 MPa, respectively. The high-stress areas in the disc exhibit a significant posterior shift, primarily distributed in the posterior-lateral region of the disc. The high-stress areas in the bilaminar region also show a posterior shift.

When perforation occurs in the anterior and middle band of the affected-side disc, there are no significant changes observed in the MVMS and stress distribution areas of both the affected-side disc and the bilaminar region compared to the anteriorly displaced disc. When perforation occurs in the medial and lateral bands of the affected-side disc, there is an increase in MVMS for the affected-side disc. The MVMS for the medial perforation is 8.2254 MPa, while the MVMS for the lateral perforation is 8.8317 MPa. However, there are no significant changes observed in the stress distribution. Compared to the anteriorly displaced side, The MVMS and stress distribution of the affected-side bilaminar region does not show any significant differences.

After perforation occurs in the posterior band of the affected-side disc, there is a significant increase in the MVMS, reaching 13.247 MPa. High-stress concentration areas are observed around the site of perforation. The MVMS in the affected-side bilaminar region also shows a significant increase, reaching 0.36855 MPa. The high-stress areas are distributed at the junction between the disc and the bilaminar region. After the occurrence of perforation at the junction between the affected posterior band and the bilaminar region, the MVMS of the affected-side disc reaches a peak value of 15.668 MPa. The stress in the bilaminar region is 0.30213 MPa, also indicating a relatively higher state. The high-stress regions in both the disc and the bilaminar region are located around the perforation site. The occurrence of perforation in the bilaminar region has a less noticeable impact on the MVMS and stress distribution of the affected-side disc. The stress in the bilaminar region is relatively high, measuring 0.25953 MPa, with the high-stress regions mainly distributed around the perforation site.

#### Stress distribution on the condyles of affected side

The results of stress distribution on the condyles of the affected side are shown in Fig. 3. C and Fig. 4. C. When the disc on the affected side is in a normal state, the MVMS experienced by the condyle is 3.445 MPa, with a uniform stress distribution and no significant stress concentration. However, after the occurrence of disc displacement, the MVMS in the affected condyle increases to 6.4715 MPa, and the stress distribution starts to shift towards the anterior part of the condyle. When perforation occurs at the junction between the disc and the bilaminar region, the MVMS in the condyle reaches its peak at 7.2234 MPa. However, no significant regular patterns of MVMS change are observed when perforations occur in other locations. After the occurrence of perforation in the posterior band and at the junction between the disc and the bilaminar region, the high-stress distribution area in the condyle becomes smaller, indicating a more concentrated stress distribution.

#### Stress distribution on the discs of normal side

**Fig. 5:**
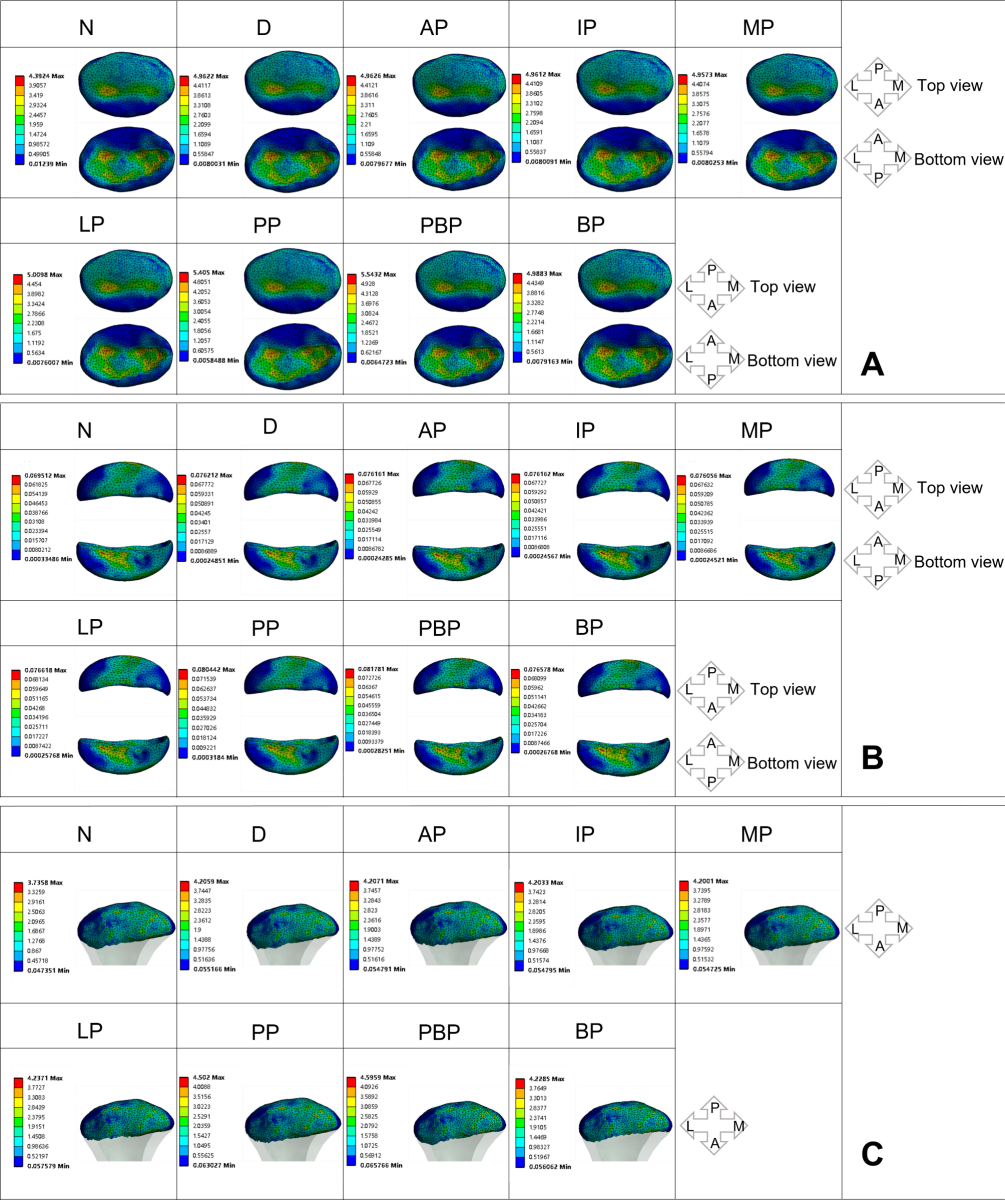
Stress distribution on normal-side TMJ impaired by disc perforated at different locations (MPa). **A**: discs, **B**: bilaminar regions, **C**: condyles

The results of stress distribution on the discs of the affected side are shown in Fig. 5. A, B and Fig. 4. D, E. In the normal state of the disc on the unaffected side, the MVMS experienced by the normal-side disc is 4.3924 MPa, while the MVMS in the bilaminar region is 0.069512 MPa. The high-stress areas in the normal-side disc are primarily distributed in the middle band and posterior band. In the normal-side bilaminar region, the high-stress areas are mainly distributed in the anterior-middle portion of the bilaminar region. After the occurrence of disc displacement on the affected side, there is a significant increase in MVMS in the normal-side disc, reaching 4.9622 MPa, and in the bilaminar region, reaching 0.076212 MPa. However, when perforations occur in the anterior band, middle band, and medial region of the affected-side disc, there are no significant changes in MVMS in the normal-side disc and bilaminar region compared to the disc displacement condition.

After the occurrence of perforation in the lateral band of the affected-side disc, there is an increase in MVMS in the normal-side disc and bilaminar region, reaching 5.0098 MPa and 0.076618 MPa, respectively. Perforation in the posterior band of the affected-side disc results in a significant increase in MVMS in the normal-side disc, reaching 5.405 MPa, and the MVMS in the normal-side bilaminar region also increases to 0.080442 MPa.

When perforation occurs at the junction between the posterior band and the bilaminar region, the MVMS in the normal-side disc and bilaminar region reach their peaks, with the disc reaching 5.5432 MPa and the bilaminar region reaching 0.081781 MPa. The impact of perforation occurring in the bilaminar region on the MVMS in the normal-side disc is not significant. When observing the influence of pathological changes in the affected-side disc on the stress distribution in the normal-side disc tissue, no significant changes in stress distribution are observed in the normal-side disc and bilaminar region.

#### Stress distribution on the condyles of normal side

The results of stress distribution on the discs of the affected side are shown in Fig. 5. C and Fig. 4. F. In the normal state of the disc on the unaffected side, the MVMS experienced by the normal-side condyle is 3.7358 MPa, with a uniform stress distribution and no significant stress concentration. After the occurrence of disc displacement, the MVMS in the affected condyle increases to 4.2059 MPa. When perforations occur in the anterior, middle, posterior, lateral, and bilaminar region of the affected-side disc, there are no significant changes in MVMS in the normal-side condyle. However, significant increases in stress are observed in the normal-side condyle when there are perforations in the posterior band and at the junction between the disc and the bilaminar region, reaching 4.502 MPa and 4.5959 MPa, respectively. When observing the influence of disc pathology on the stress distribution in the normal-side condyle, no significant changes in stress distribution are observed in the normal-side disc and bilaminar region.

### The impact of different sizes of perforations of the disc on TMJ stress

#### Stress distribution on the affected TMJ

**Fig. 6:**
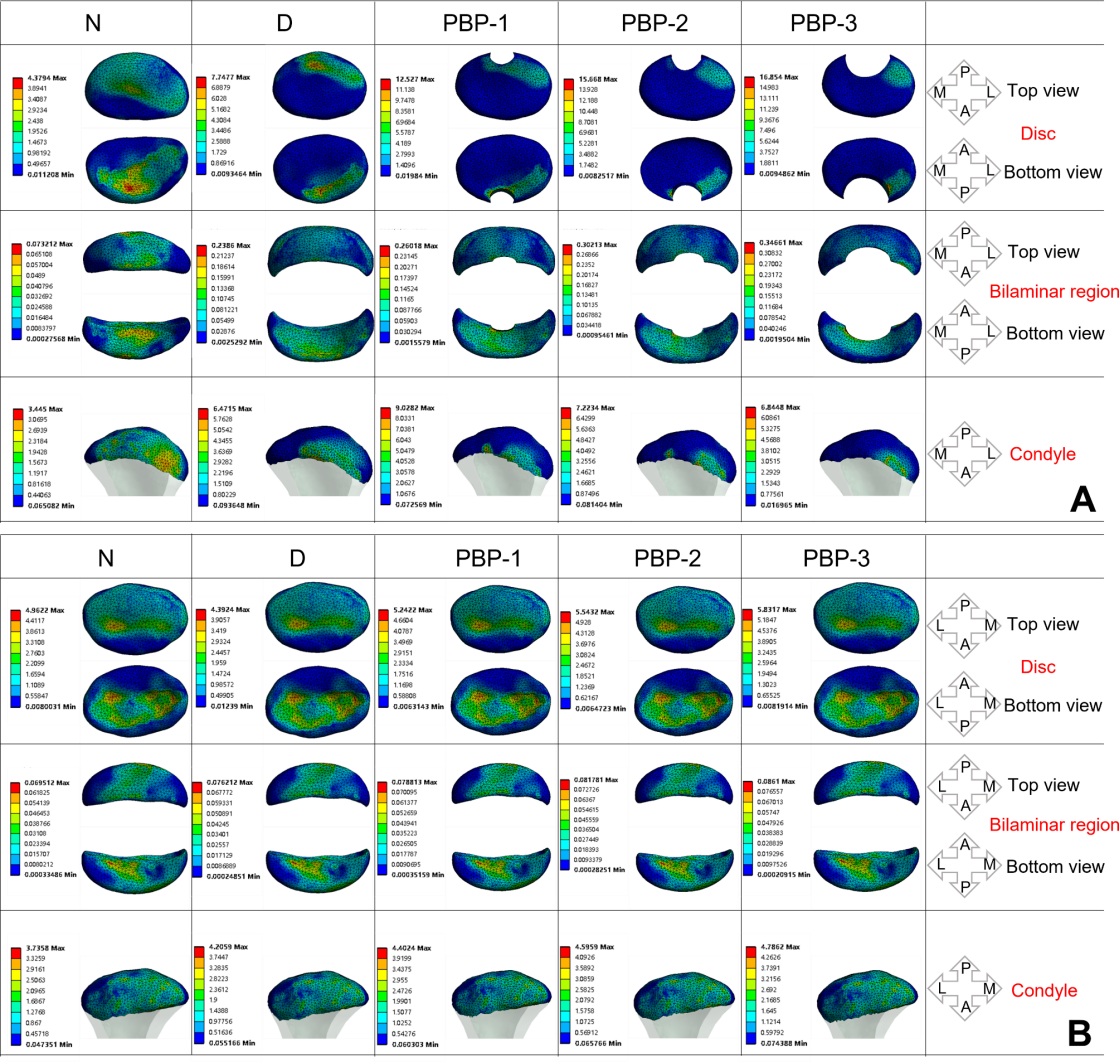
Stress distribution of the affected-side TMJ (**A**) and normal-side TMJ (**B**) impaired by disc perforated with different apertures (MPa)

**Fig. 7:**
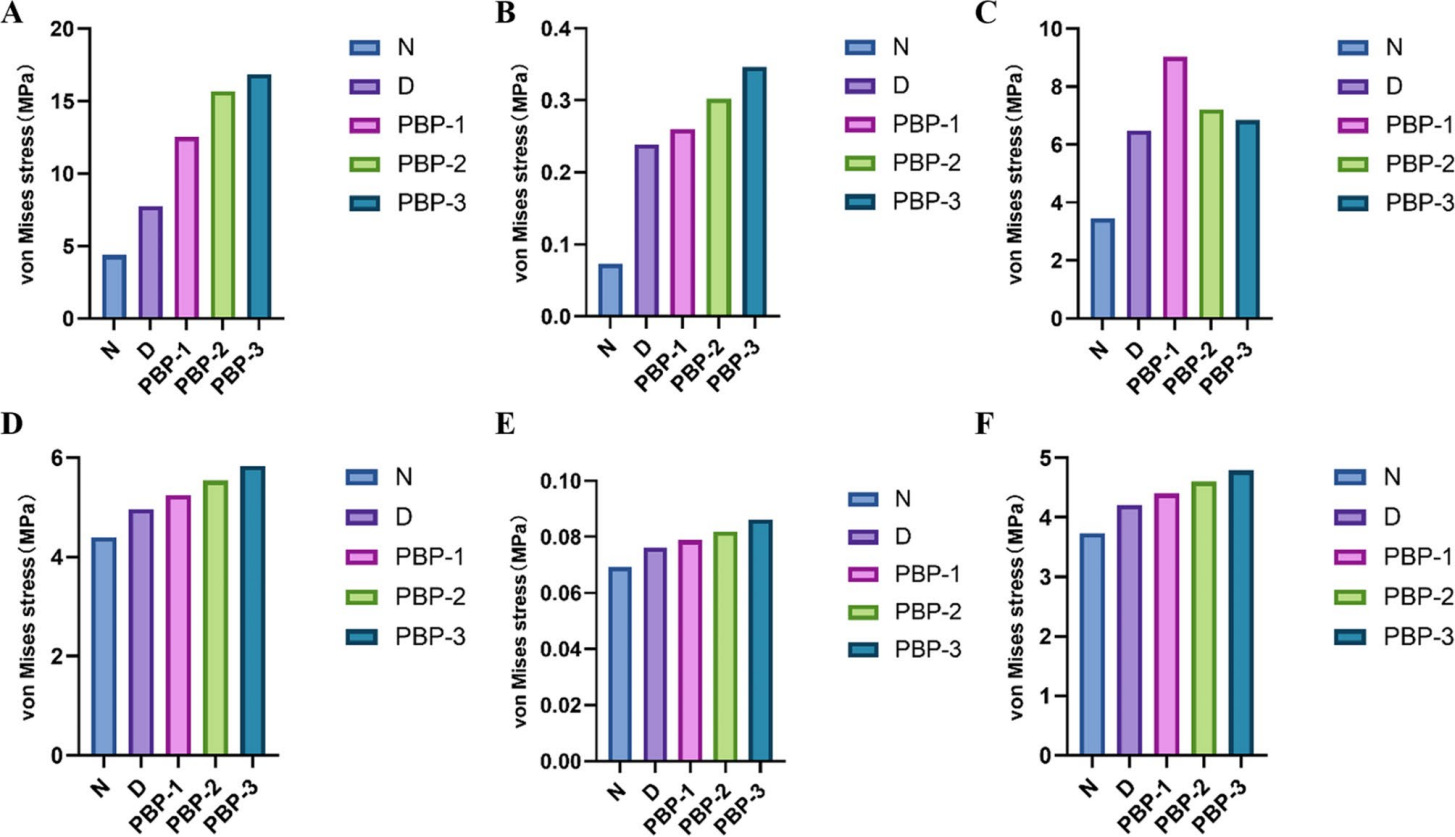
Peak value of the stress on the TMJ affected by perforated disc with different apertures (MPa). **A**: affected-side discs; **B**: affected-side bilaminar regions; **C**: affected-side condyles; **D**: normal-side discs; **E**: normal-side bilaminar regions; **F**: normal-side condyles

The results of stress distribution on the TMJ of the affected side are shown in Fig. 6. A and Fig. 7. A, B, C. As the disc undergoes displacement accompanied by an increasing perforation size, the MVMS in the affected-side disc and bilaminar region gradually increases. At the 1/2 perforation size, the stress in the disc and bilaminar region reaches its peak, measuring 16.854 MPa and 0.34661 MPa, respectively. The stress distribution area also decreases with the expansion of the perforation size, leading to significant stress concentration, particularly around the perforation site.

However, the MVMS in the condyle does not increase proportionally with the perforation size. As the affected-side disc progresses from a normal state to anterior displacement and further to the 1/4 perforation stage, the MVMS in the condyle gradually increases. At the 1/4 perforation, the condyle reaches its peak MVMS, measuring 9.0282 MPa. However, as the perforation size expands to 1/3 and 1/2, the maximum stress in the condyle shows a continuous decrease. These findings indicate that as the perforation size increases, the MVMS in the affected-side disc and bilaminar region progressively rises, reaching a peak at the 1/2 perforation size. Meanwhile, the condyle’s maximum stress varies during disc displacement and perforation progression, peaking at the 1/4 perforation size and decreasing thereafter when the perforation size expands to 1/3 and 1/2.

#### Stress distribution on the normal TMJ

The results of stress distribution on the TMJ of the affected side are shown in Fig. 6. B and Fig. 7. D, E, F. On the normal side, the MVMS experienced by the articular disc, bilaminar region, and condyle all increase continuously with the expansion of the perforation size in the articular disc. The stress peaks are measured at 5.8317 MPa for the articular disc, 0.0861 MPa for the bilaminar region, and 4.7862 MPa for the condyle. However, the stress distribution in these structures does not show significant changes despite the increase in MVMS.

## Discussion

DP is a progressive degenerative condition [20, 21]. DP leads to a change in the stress environment of the TMJ, and clinically, there are also cases of condylar bone remodelling that occur after DP [6, 22]. Therefore, studying the biomechanical behaviour of TMJ is of significant importance for understanding the aetiology of TMJ disorders. In this study, we constructed a 3D FE model of the TMJ with DP and performed static analysis under the ICP state. We systematically investigated the effects of disc displacement and perforation on the stress distribution in the TMJ. Our study demonstrated that anterior displacement and perforation of the disc have adverse effects on the TMJ.

In this study, after the anterior displacement, there is a significant increase in the maximum stress experienced by both sides of the disc, condyles, and bilaminar region. The primary stress distribution area of the affected-side disc shifts to the posterior-lateral region, consistent with A’. Pérez del Palomar and Long’s Clinical study [23, 24]. It validates the effectiveness of the FEM and provides strong evidence for the predictive and guiding role of FE analysis in TMJ-related research. When perforations occur at the junction of the posterior band and the bilaminar region, the MVMS of all bilateral TMJ structures is significantly higher than perforations in other areas. This indicates that the perforations occurring in the posterior band and at the junction pose greater harm to bilateral TMJ structures than perforations in other areas. Currently, conservative treatment remains the primary approach for treating DP, and there is no specific indication for surgical intervention [25, 26]. According to the findings of this study, DP occurring in the posterior band and at the junction of the posterior band and the bilaminar region may be more suitable for early surgical treatment to prevent destructive damage to the disc. When the perforation continues to enlarge, the MVMS experienced by the bilateral discs, bilaminar regions, and the condyles of the unaffected side increases as the perforation of the affected-side disc expands. Moreover, the MVMS is predominantly located around the perforation site. We speculate that this stress distribution trend leads to further enlargement of the perforation lesion. This indicates that perforations occurring at the posterior band and the junction should be addressed and treated as early as possible. However, we also observed that the stress on the condyle of the affected side does not follow this pattern. It is important to note that our sample size was small, and the observations were limited to the head of the condyle. This pattern needs further confirmation through future studies. It was also found that the displacement of the joint disc and changes in the location and size of perforations not only affect the stress on the affected-side TMJ but also result in changes in stress on the normal-side TMJ. Lai et al.‘s study also revealed that an increase in friction coefficient can influence the stress on the normal-side TMJ, aligning with the findings of this study [27]. Therefore, in the treatment of TMD, it is essential to consider not only the treatment of the affected side but also to take preventive measures for the unaffected side to prevent bilateral TMJ pathologies and minimize the suffering of the patients.

This study established a relatively detailed FE model of the TMJ, including the human masticatory system, and for the first time validated the impact of the location and size of DP on the TMJ. However, the FE model was reconstructed based on only one participant, and the presented results lack statistical value. There may be variations in the results due to differences in TMJ morphology and the degree of disc displacement. Additionally, limited by software and imaging equipment, we only conducted static analysis in the closed-mouth position, which cannot dynamically represent the patient’s jaw movement. Furthermore, bone and teeth are not homogeneous materials, and Zheng’s study has also demonstrated that different regions of the bone have different Young’s modulus and Poisson’s ratio [9]. The results obtained from heterogeneous models may differ from those obtained from homogeneous models, which should be taken into account and improved in future research.

## Conclusion

1. Unilateral disc anterior displacement results in an increased stress on both TMJs.
2. Unilateral DP further affects the stress on both sides of the TMJs.
3. The enlargement of the DP aperture leads to a further increase in stress on both temporomandibular joints.
4. TMD is a progressive condition, and timely intervention is necessary in the early stages to prevent the worsening of the condition.

## Data Availability

All data were calculated by the software. The datasets used and/or analyzed during the current study are available from the corresponding author on reasonable request.

## Abbreviations

TMJ: temporomandibular joint
DP: disc perforation
TMD: temporomandibular disorder
FEM: finite element method
ICP: intercuspal position
MVMS: maxim von Mises stress
